# Long-Term Release of Dexamethasone With a Polycaprolactone-Coated Electrode Alleviates Fibrosis in Cochlear Implantation

**DOI:** 10.3389/fcell.2021.740576

**Published:** 2021-10-29

**Authors:** Dongxiu Chen, Yanjing Luo, Jing Pan, Anning Chen, Dong Ma, Muqing Xu, Jie Tang, Hongzheng Zhang

**Affiliations:** ^1^Department of Otolaryngology Head and Neck Surgery, Zhujiang Hospital, Southern Medical University, Guangzhou, China; ^2^Hearing Research Center, Southern Medical University, Guangzhou, China; ^3^Key Laboratory of Biomaterials of Guangdong Higher Education Institutes, Department of Biomedical Engineering, Jinan University, Guangzhou, China; ^4^Department of Physiology, School of Basic Medical Sciences, Southern Medical University, Guangzhou, China; ^5^Key Laboratory of Mental Health of the Ministry of Education, Southern Medical University, Guangzhou, China

**Keywords:** cochlear implantation, fibrosis, PCL, inflammation, TGF-β1

## Abstract

Cochlear implantation (CI) is the major treatment for severe sensorineural hearing loss. However, the fibrotic tissue forming around the electrodes reduces the treatment effectiveness of CI. Dexamethasone (DEX) is usually applied routinely in perioperative treatment of cochlear implantation (CI), but its diffusion in the inner ear after systemic administration is limited. In the present study, an electrode coated with polycaprolactone (PCL) loaded with dexamethasone was developed with a simple preparation process to maintain the stability of the electrode itself. The DEX-loaded PCL coating has good biocompatibility and does not change the smoothness, flexibility, or compliance of the implant electrode. Stable and effective DEX concentrations were maintained for more than 9 months. Compared with the pristine electrode, decreasing intracochlear fibrosis, protection of hair cells and spiral ganglion cells, and better residual hearing were observed 5 weeks after PCL-DEX electrode implantation. The PCL-DEX electrode has great potential in preventing hearing loss and fibrosis by regulating macrophages and inhibiting the expression of the fibrosis-related factors IL-1β, TNF-α, IL-4, and TGF-β1. In conclusion, the PCL-DEX electrode coating shows promising application in CI surgery.

## Introduction

Cochlear implantation (CI) is the main treatment for severe sensorineural hearing loss (Li et al., 2017). This procedure requires the insertion of electrodes into the cochlea, by which electrical signals containing external sound information are delivered to cochlear neurons, enabling hearing restoration in patients with sensorineural deafness. Over 5% of the world’s population—or 466 million people have disabling hearing loss (432 million adults and 34 million children). The long-term efficacy of CI is critical for patients.

However, there are still many problems in CI that need to be improved. Among them, fibrotic tissue forming around the electrodes normally decreases the curative effect of CI. Autopsy pathology studies on human temporal bone specimens of CI recipients found that fibrotic tissue existed in most cochleae (Seyyedi and Nadol, 2014; O’Malley et al., 2017) and fibrous sheaths were usually formed around the electrode in the implanted cochleae (Ishai et al., 2017). A dense fibrotic sheath increases the impedance of the electrode array, resulting in unstable electrical signals and increasing power consumption (Clark et al., 1995; Sanderson et al., 2019). A tracking of 85 cases showed that various degrees of hearing loss occurred in 38% of patients 5 years after CI surgery (Rowe et al., 2016; Foggia et al., 2019). The progressive loss of CI restored hearing was not significantly related to electrode type or the insertion method (*via* round window or cochlear fenestration) but was related to the changes in electrical impedance, which was probably induced by fibrosis around the electrode array (Clark et al., 1995).

Moreover, severe fibrosis in the cochlea causes trauma to the fine structure in the cochlea and blocks the sound vibration conducting to the cochlear basilar membrane (Choi and Oghalai, 2005). Fibrosis in the cochlea is one of the main reasons for the loss of residual hearing after CI, affecting the availability of an acoustic-electricity combined stimulation model in the rehabilitation of CI. In addition, fibrosis in the cochlea makes it difficult and risky to reimplant the electrode in the cochlea (Côté et al., 2007). In many second implantation operation cases, fibrosis in the scala tympani impedes the insertion of electrode arrays and even causes surgical failure. Therefore, a strategy reducing the formation of fibrosis is urgently needed to improve the long-term effect of CI (Eshraghi et al., 2020).

Clinically, glucocorticoids, such as DEX, are applied systemically or locally to reduce the inflammation and foreign body response of CI. However, due to the lack of stable and efficient delivery of drugs in the cochlea or significant side effects of long-term systemic treatment, the current clinical treatment effects vary and are not obvious, limiting the usage of glucocorticoid treatment (Cortés Fuentes et al., 2020).

In many previous studies, multiple bioactive materials and coatings have been applied to preserve the neural function and reduce the inflammation and fibrosis (Liu et al., 2018; Shang et al., 2018; Guo J. H. et al., 2020; Guo et al., 2021; Han et al., 2020; Xia et al., 2020; Zhao et al., 2020; Yang et al., 2021). In the present study, we proposed a drug-loaded electrodes for CI. We employed polycaprolactone (PCL), a polymer that has been approved for clinical use, for long-term release of loaded DEX by coating on CI electrodes (Gaharwar et al., 2014; Zhou et al., 2018). Our data showed that the PCL-DEX coating significantly attenuated inflammation and the formation of fibrosis after CI in rats, preventing the progressive loss of residual hearing induced by fibrosis in the cochlea. Our study suggests that polycaprolactone is a promising material for the long-term release of loaded drugs into the cochlea by coating implants in CI.

## Materials and Methods

### Electrode Coating

Silicone dummies (MEDEL, Innsbruck, Austria) 10 mm in length were used for preparing coating electrodes for cochlear implantation, with marker at 3 and 6 mm, measuring 0.2 mm in diameter at the tip and 0.3 mm at a position 6 mm from the tip. The polycaprolactone (PCL) pellets (AiKe, China) and dexamethasone powder (DEX, purity ≥ 98%) (Meryer, Shanghai, China) were mixed in different ratios (95:5, 90:10, and 80:20). The mixture was then dissolved in dichloromethane [10% (w/w)] (Aladdin, China) at RT (room temperature, 27°C). Then the PCL-DEX copolymer was coated on the tip of silicone dummy by dipping the dummy (∼10 mm) into the mixture for 10 s for one time (Luo et al., 2021). The coated electrodes were dried at RT for 24-h for further characterization. The coating thickness was measured by an optic microscope. The loading capacity was determined as following:


The⁢drug⁢loading⁢capacity=

$$\frac{\mathrm{weight}\,\mathrm{of}\,\mathrm{DEX}\,\mathrm{in}\,\mathrm{the}\,\mathrm{coating}}{\mathrm{weight}\,\mathrm{of}\,\mathrm{PCL}\,\mathrm{and}\,\mathrm{DEX}\,\mathrm{in}\,\mathrm{the}\,\mathrm{coating}}\,\mathrm{100}\%$$

### Scanning Electron Microscopy

The coated electrodes were sputter-coated with thin layers of gold, and the surface morphologies of the coated electrodes were examined by SEM (Ultra55, Zeiss, Germany).

### Tensile Testing

To determine effects of PCL coating on the flexibility of the coated electrodes, tensile testing was performed by an electronic universal testing machine (BL-GRW005 K, Zwick/Roell, Germany). The electrode/dummy length was set as 70 mm, and the crosshead speed was 1 mm/min. The displacement-pressure curves were recorded and mean values were calculated.

### Dexamethasone Release Measurement

Silicone dummy (35 mm long with an 8 mm diameter) coated with PCL-DEX copolymer was soaked in 3.8 mL artificial perilymph (1.3 CaCl_2_, 1.8 MgCl_2_, 5.4 KCl, 137 NaCl, 5 glucose, 5 HEPES, in mmol/L) at 37°C, allowing the DEX released from the copolymer. Aliquots (540 μL) of artificial perilymph were taken out to measure the DEX concentration from 1 h to 270 days. After each measurement, the same volume of fresh artificial perilymph was replenished. DEX mass and concentration in artificial perilymph were determined using a high- performance fluid chromatography (HPLC) (Agilent 1260, United States) at 275 nm.

### Cell Culture and Viability Assays

L929-T-25mice fibroblasts cells and House Ear Institute-Organ of Corti 1 (HEI-OC1) cells were used to examine the biocompatibility of the PCL-DEX copolymer. The 35 mm petri dishes were pre-coated with PCL. Cells were cultured in 35 mm Petri dishes with/without PCL-DEX coating in a CO_2_ incubator. L929 cells were cultured in RPMI-1640 complemented with 10% FBS (Gibco, United States), 5% CO_2_ at 37°C. HEI-OC1 cells were cultured in DMEM medium (Gibco, United States) complemented with 10% FBS, 10% CO_2_ at 33°C. After 48 h, the cell densities were calculated under an optic microscope.

For cell viability assays, L929 cells or OC1 cells were seeded into 96-well plate at 5 × 10^3^ cells per well for 24 h. For preparation of leached solution, 0.1 wt% DEX and 1 cm long electrodes coated with PCL, PCL- 5%DEX, PCL- 20%DEX were, respectively, immersed in culture media for 24 h. The leached solution were collected and sterile filtered using a filter of 0.22 μm pore. The culture medium was replaced with leached solution or normal fresh culture medium. 24 h later, a CCK-8 kit (Beyotime, Shanghai, China) was used to examine the cell viability by following the standard protocol.

### Animal Surgery

CIs were performed monaurallyin normal hearing SD rats aged 5–7 weeks. Rats were anesthetized with an intraperitoneal injection of pentobarbital sodium (45 mg/kg). Additional doses were given as needed. A retroauricular incision was made, and the nearby muscles and skin were separated to expose the bulla. The bulla was opened under a microscope using a delicate drill. The round-window membrane was then incised with a bent forceps. The electrode was gently inserted through the incision into the scala tympani, to a depth of 3 mm. In DEX group, 0.1 mL of DEX (5 mg/mL) was injected into the cochlea in the scala tympani after insertion of pristine electrodes. In sham group, no electrode was implanted and all other surgical operations remained consistent with the implanted groups. The round window was patched with a small piece of muscle in case of sustained leaking of perilymph. Mobility and integrity of the ear drum and ossicular chain was monitored by microscopic examination during all stages of the surgery. After the surgery, wounds were carefully sutured and animals were sent back to their home cages.

### Auditory Brainstem Response

Auditory brainstem response was examined to evaluate hearing function as described in our previous study (Hang et al., 2016). In brief, anesthetized rat (pentobarbital sodium, 45 mg/kg, i. p.) was placed on a vibration isolation table in an electromagnetically and acoustically shielded room. Three subcutaneous needle electrodes were placed on the vertex (recording) of skull, the loose skin behind the tested ears (reference) and the opposite ears (ground). 5 ms tone bursts with different frequency (2, 4, 8, 16, 24, and 32 kHz) and intensity were generated by the software SigGenRP and delivered by a speaker placed 15 cm away from the ear at a rate if 10/s. The ABR waveforms were amplified, recorded, averaged, and stored by a software BioSigRZ (Tucker-Davis Technology, Alachua, FL, United States). During recording, the contralateral ear (non-operated) was plugged with a silicon putty earplug (Mack’s, United States). The amplified evoked responses with 256 sweeps were averaged in real time. The ABR thresholds were defined as the highest sound level at which no response was detectable. After recovery from the anesthesia, animal was sent back to its home cage.

### Histology and Immunostaining

After the last ABR recording, the animal was scarified and the cochlea was dissected. The cochlea was fixed by 4% paraformaldehyde at 4°C overnight and decalcified for 10 days. The organs of Corti (OC) were isolated and cut evenly into three pieces for immunostaining. To quantify the fibrosis size, the cochlea was sectioned serially in the axial plane at a thickness of 20 μm. Usually, 50–100 slides containing fibrosis were collected and photoed under a Nikon optical microscope. The outline of the fibrous tissue and the scala tympani were tracked by the software Image-Pro Plus. The whole volume of fibrous tissue and scala tympani were calculated by these slides, and the percentage of fibrous tissue in scala tympani was determined. Sections from the modiolar plane were stained with an H&E kit (Beyotime, Shanghai, China) using standard protocols.

Representative sections and OCs were used for immunofluorescence staining. Samples were permeabilized by 0.3% Triton X-100 in PBS (Sigma, United States) and blocked with 10% normal goat serum (Sigma, United States) for 1 h at RT. Sections or tissues were incubated with primary antibodies α-SMA (1:1,000, Sigma, United States), type I collagen (1:1,000, Sigma, United States), Map2 (1:1,000, Protein Tech, Chicago, IL, United States), Myosin 7a (1:1,000, Protein Tech, Chicago, IL, United States), and CD68 (1:1,000, Sigma, United States). After the primary antibodies were completely washed out with PBS, samples were incubated with Alexa Fluor 568-conjugated or Alexa Fluor 488-conjugated secondary antibody (BBI Life Science, China) for 2 h at RT. The fluorescence images were acquired using a confocal laser scanning microscopy (Nikon, Japan) at 20× magnification.

### Western Blotting and ELISA Measurement

The protein extracted from the whole cochlea was used for western blotting. 30 μg protein sample was separated by SDS–PAGE gels and transferred to polyvinylidene fluoride (PVDF) membrane (Millipore, Bedford, MA, United States). After blocking with 5 % non-fat milk in TBS-T (Beyotime, Shanghai, China) for 2 h at RT, the membrane was incubated with the primary antibodies α-SMA (Sigma, United States), type I collagen (Sigma, United States), TGF-β1 (Abcam, England), or TNF-α (Abcam, England) overnight at 4°C. The PVDF membrane was washed and incubated with secondary antibodies conjugated to the HRP (BBI Life Sciences, Shanghai, China) for 2 h. Protein bands were detected with a chemiluminescence detection system (Image 600, GE Healthcare, Chicago, IL, United States). Protein grayscale was analyzed with software Image Quant TL (GE Healthcare, Chicago, IL, United States).

The levels of IL-1β and IL-4 released in cochleae after electrode implantation were measured using an ELISA kits (Abcam, United States). The perilymph and tissue lysate were collected from the cochlea. For each group, data was calculated as percentage compared to the level in the control groups.

### Data Analysis

The data analysis was performed using SPSS 22.0 (IBM, United States). The quantitative analyses were independently performed by two of the authors in a blind manner to ensure the consistency across all data. Student’s *t*-test was performed for the comparison of two groups and comparisons among multiple groups were analyzed by analysis of variance (ANOVA) followed by multiple comparison tests for *post hoc* analysis. Significance was defined as *p* < 0.05. GraphPad Prism 7 (GraphPad Software, SanDiego, CA, United States) was used for was used for statistical analyses and graphical display.

## Results

**FIGURE 1:**
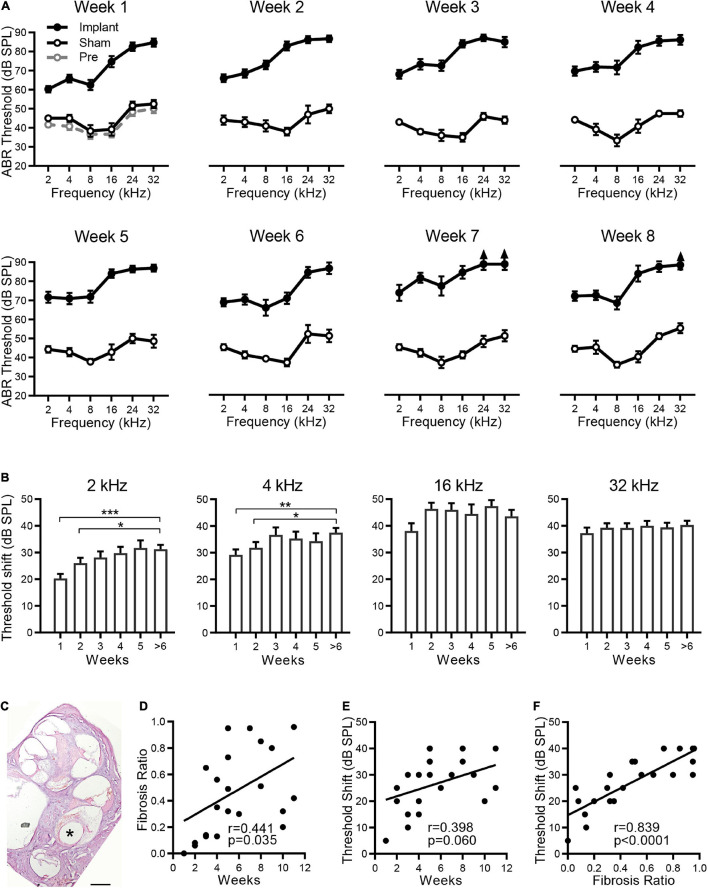
Hearing loss is associated with fibrosis after CI. **(A)** The effects of electrode implantation on the ABR thresholds at different implantation times. The ABR threshold-frequency curves of the implant (filled circles) and sham groups (open circles) were plotted in the same panel from week 1 to week 8. For the implant group, *n* = 18, 20, 21, 18, 21, 7, 5, and 11 for weeks 1 to 8, respectively. For the sham group, *n* = 6 for each week. The ABR thresholds before implantation (Pre-Op, gray open circles, *n* = 6) are plotted in the panel of week 1. The data are presented as the mean ± SE. For some data points, the arrows indicate the thresholds above the limit of measurement (90 dB SPL). **(B)** ABR threshold shifts at 2, 4, 16, and 32 kHz versus the sham group at different implantation times. Data are presented as the mean and SE. *p < 0.05; **p < 0.01; ***p < 0.001. *n* = 18, 20, 21, 18, 21, and 23 for weeks 1, 2, 3, 4, 5, and > 6, respectively. **(C)** Optical microscope image of a representative H&E-stained cross section of a rat cochlea implanted with electrode for 5 weeks. “*” indicates the trajectory of the electrode insertion. Scale bar = 500 μm. The percentage of fibrous tissue in scala tympani was determined as fibrosis ratio. **(D,E)** The pooled data show the proliferative tissue volume **(D)** and the ABR threshold shifts at 2 kHz **(E)** distributed over the implantation time. **(F)** The relationship between ABR threshold shifts and proliferative tissue volume. The line indicates the linear correlation coefficient.

To evaluate the postimplantation effects on the hearing of the implanted ear, silicone dummies were implanted into the rat cochlea, and the ABRs were measured from the implanted ear before and after implantation. As shown in Figure 1A, compared with the ABRs before implantation (pre., gray dashed line and open circles), no significant ABR threshold shift was observed at any sound frequency in the sham group (sham, black line and open circles) 1 week after the operation (*p* > 0.05, ANOVA). The ABR thresholds remained unchanged in the sham group from week one to week eight (*p* > 0.05, ANOVA). For the silicone electrode implantation group (implant, black lines and filled circles), the ABR thresholds at all sound frequencies were significantly elevated from the first week after implantation (vs. sham group, *p* < 0.001, ANOVA), indicating the impairment in hearing of pristine electrode insertion. However, the time course of ABR threshold changes was diverse for different sound frequencies. For tones of 2 and 4 kHz, the ABR thresholds increased progressively with implantation time and reached the maximal threshold shift at week 3 after implantation (Figure 1B). The ABRs for 2 and 4 kHz tones were responded to by the apical portion of the cochlea; therefore, these threshold shifts indicate progressive functional impairment of the apical cochlea by implantation. However, the cochlear implant resulted in severe hearing loss for 16 and 32 kHz tones with a mean ABR threshold shift > 35 dB from the first week after implantation (Figure 1B), implying a possible impairment at the high-frequency end of the cochlea by implant. In the following experiments, the ABR threshold at 2 kHz was chosen to evaluate the chronic effects of electrode implantation.

The cochlear implant causes a foreign body response with associated fibrosis. As shown by a representative cochlear cross section in Figure 1C, significant fibrous tissue was found around the electrode 5 weeks after implantation. By measuring the volume of fibrous tissue and scala tympani of each implantation, we calculated the percentage of scala tympani occupied by the fibrosis to evaluate the extent of fibrosis. Pooled data showed that fibrosis varied greatly in different individuals, and only a weak correlation was found between the size of fibrosis and the implantation time (*r* = 0.441, *p* = 0.0352, Figure 1D). Moreover, no correlation was observed between the ABR threshold shifts (at 2 kHz) and the implantation time (*r* = 0.398, *p* = 0.0599, Figure 1E). These results suggest that the implantation time may not be the key factor causing fibrosis and hearing loss. Further analysis showed that this ABR threshold shift increased with the size of fibrosis (*r* = 0.839, *p* < 0.0001, Figure 1F), and their strong relationship indicates that fibrosis is the major pathogenetic factor in the hearing loss observed after cochlear implantation.

**FIGURE 2:**
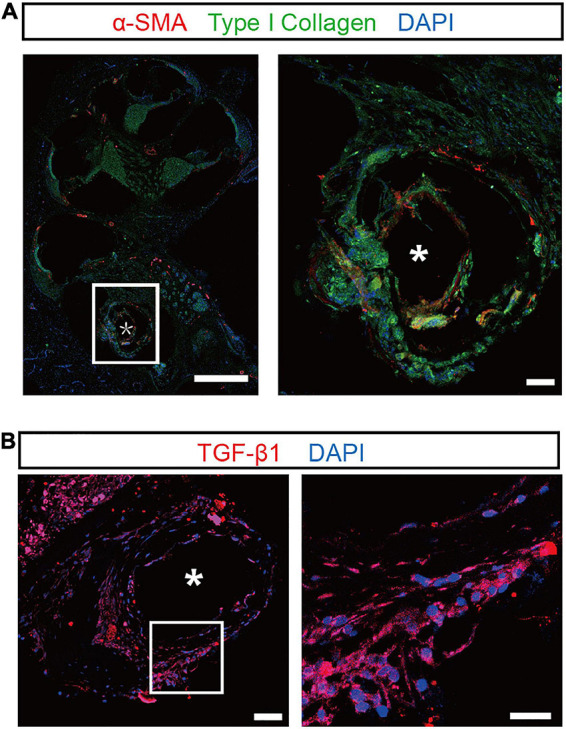
Confocal images of the proliferative tissue after electrode insertion in the cochlea. **(A)** Representative immunostaining images showing the proliferative tissue in the tympanic scala 5 weeks after implantation. α-SMA (red) and type I collagen (green) were evident in the proliferative tissue (magnified view, right panel). Scale bar = 500 and 50 μm for the left and right panels, respectively. **(B)** Left panel: representative immunostaining images showing the expression of TGF-β1 (red) in the proliferative tissue 5 weeks after implantation. Scale bar = 50 μm. Right panel: the enlarged plot of the white box in the left panel. Scale bar = 50 μm. “*” indicates the trajectory of the electrode insertion.

The tissue responses to silicone dummies were assessed by immunostaining of α-SMA, type I collagen, and TGF-β1 in proliferative tissue. α-SMA and type I collagen are markers of myofibroblasts and major components of the extracellular matrix in fibrotic tissue, respectively. As shown in the representative images, robustα-SMA immunoreactivity was observed in the proliferative tissue surrounding the implant, and strong type I collagen expression was distributed throughout the proliferative tissue (Figure 2A). Moreover, immunoreactivity of TGF-β1, which regulates the proliferation and differentiation of myofibroblasts and promotes the secretion of extracellular matrix, was also found in proliferative tissue surrounding the implant and in generation of fibrosis (Figure 2B). These results confirmed that proliferative tissue is the typical fibrous tissue in our experiments.

**FIGURE 3:**
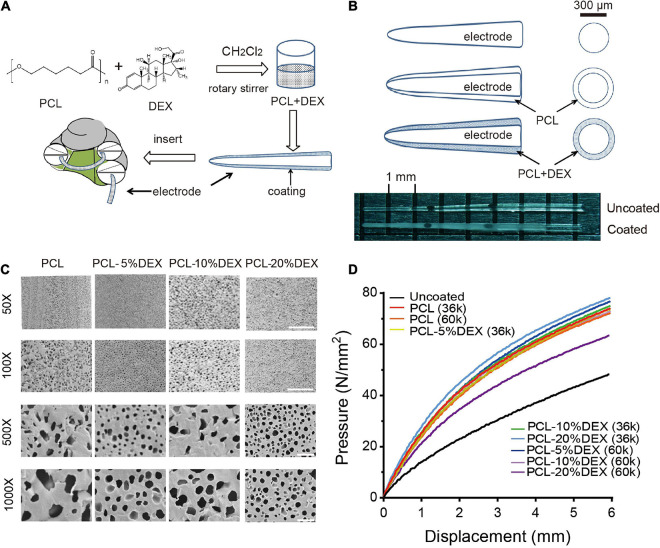
Cochlear implant PCL-coated electrodes and their physical properties. **(A)** Schematic showing the PCL-DEX coating process of the cochlear implant electrode. **(B)** Top panels show diagrams of the electrode and its coating. Bottom photographs show the tips of uncoated (pristine) and PCL-DEX-coated electrodes. **(C)** Scanning electron microscopic images show the surface of electrodes coated with PCL, PCL-5%DEX, PCL-10%DEX, and PCL-20% DEX (MW = 36 k). Scale bar = 100, 20, 10, and 5 μm for 50×, 100×, 500×, and 1,000× magnifications, respectively. **(D)** Color coded lines show the pressure produced by the pristine and coated electrodes at different bend displacements.

Currently, the therapeutic method to treat fibrosis after cochlear implantation in clinical practice is administration of dexamethasone. However, side effects of systemic administration limit long-term application. The synthesized PCL-DEX was designed to achieve sustained drug release and maintain protective DEX concentrations as the coating of the electrode implanted into the cochlea along with the electrode (Figure 3A). The thickness of the coating was also determined to be 48.66 ± 0.35 μm by optical microscopy (Figure 3B).

In the present study, 5, 10, and 20% DEX were used to prepare the PCL-DEX electrodes. The microstructure of the PCL (MW = 36 k) coating surface was examined by SEM. As shown by the SEM images in Figure 3C, a smooth appearance was observed in PCL coating surfaces with and without DEX loaded. High magnification images revealed the fine texture of the surfaces, and pore-like apertures were observed. DEX was contained in these apertures and released when PCL was degraded slowly in the cochlea. Abundant apertures with homogenic and small sizes are instrumental in the steady and sustained release of DEX.

Good mechanical flexibility is required when PCL-coated electrodes are inserted into the cochlear coil. Tensile displacement was applied to determine the flexibility of PCL-coated electrodes with two different PCL molecular weights (MW = 36 and 60 k). At the maximal bending displacement, no cracking in the PCL coating was observed for any electrode tested. The flexibility of all PCL-DEX electrodes was acceptable for cochlear implantation compared to pristine electrodes (Figure 3D). These images suggest that the PCL-DEX electrode prepared with 20% DEX may achieve sufficient and steady DEX release. These results indicate that the physical features of PCL-DEX-coated electrodes meet the requirement of cochlear implantation.

**FIGURE 4:**
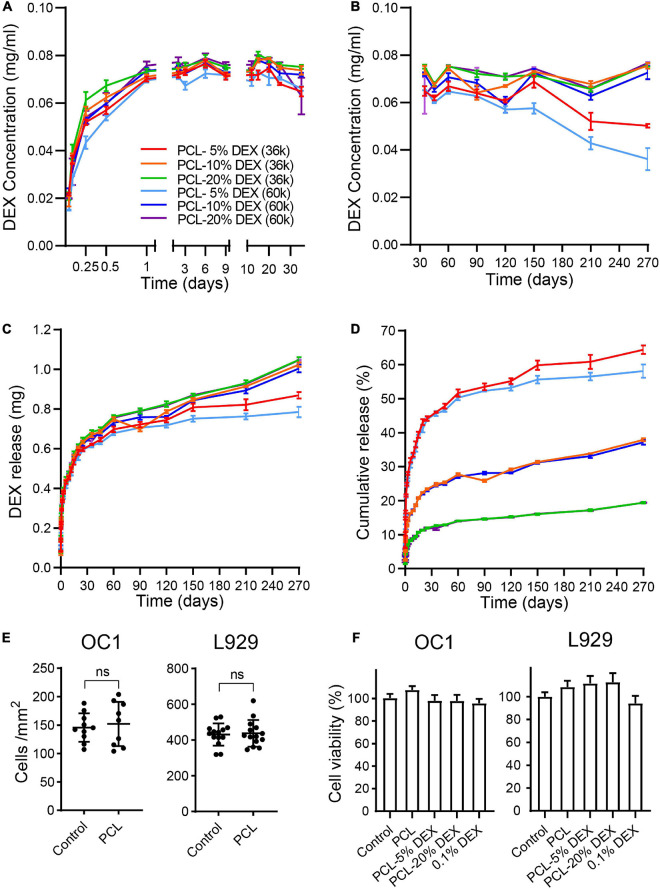
The release of DEX. **(A,B)** The concentration of dexamethasone released by PCL-DEX-coated electrodes in artificial perilymph fluids each day in the short term [**(A)** within 30 days] and long term [**(B)** 30–270 days]. Color-coded lines indicate the electrodes coated with different molecular weights of PCL (MW36k or 60k) containing DEX from 5 to 20%. **(C)** Cumulative mass of DEX released by different PCL-DEX-coated electrodes in 3.6 ml of artificial perilymph fluids. DEX mass was measured from day 0 to 270. **(D)** The percentage of total DEX loaded in PCL released from day 0 to day 270 for different PCL-DEX formulas. The color code fits **(A–D)**. The data are presented as the mean ± SE. *n* = 10 for each group. **(E)** Cell density of HEI-OC1 cells and L929 cells cultured in PCL-coated and noncoated (control) plates. The data are presented as the mean ± SE. ns: no significant difference, *p* > 0.05, *n* = 10 for each group. **(F)** The cell viability of HEI-OC1 and L929 cells cultured in normal medium (control) and leach solution of PCL, PCL-5%DEX, PCL-10%DEX, and PCL-20% DEX and 0.1%DEX. The data are presented as the mean ± SE. *n* = 13 for OC1 cells and *n* = 23 for L929 cells.

The release properties of the PCL-DEX electrodes were further evaluated by soaking PCL-DEX electrodes in artificial perilymph to test the DEX concentration by HPLC. DEX was released very quickly and reached a stable concentration within 1 day. The stable concentration was maintained for 270 days (Figures 4A,B). DEX was released in a slow rate. Slow and sustained release of DEX can lead to enhanced potency (Figure 4C). To evaluate the DEX loading capacity in PCL-DEX electrodes, the cumulative amount of DEX released and the percentages in total DEX loading were counted. The PCL-20%DEX (MW36k) electrode had the most cumulative amount of DEX (1.05 mg ± 0.02 mg) released in 270 days and the lowest percentage (19.4 ± 0.42%) in total DEX carried by PCL carried by PCL (Figures 4C,D). And for PCL-20%DEX electrode, stable and effective DEX concentrations (0.073 ± 0.002 mg/mL) had been maintained for more than 9 months. Combining clinical needs, the PCL-20%DEX electrodes were chosen for implantation (Figure 4D). This result indicated that the drug loading content was sufficiently adequate to achieve sustained and effective release. DEX was completely encapsulated in the PCL coating, which slowly released DEX for approximately 270 days at relatively stable concentrations.

HEI-OC1 cell line, which derived from the conditionally immortalized mouse auditory cells, were commonly used cell line in many previous reports (He et al., 2017, 2021; Li et al., 2018; Zhang et al., 2019, 2021). Here HEI-OC1 cells and L929 cells were cultured on the PCL-coated surface to confirm the biocompatibility of our coating. No significant difference in the cell density was observed on the PCL surface compared to that of the normal culture dish for either cell type (Figure 4E, *p* > 0.05, Student’s *t*-test). The effects of PCL loaded with different DEM concentrations on cell viability were then examined by using the CCK-8 assay. Compared with the cells cultured in normal culture medium, no significant difference in cell viability was found for either cell type cultured in the soaking solution of PCL, PCL-5% DEX, PCL-20% DEX, and culture medium containing 0.1% DEX (Figure 4F, *p* > 0.05, one-way ANOVA). Together with the physical feature data, these results indicate that our PCL-DEX coating is safe and suitable for cochlear implantation. The formula of 20% DEX loaded with 36 k MW PCL (denoted as PCL-DEX hereafter) was selected for the following *in vivo* experiments.

**FIGURE 5:**
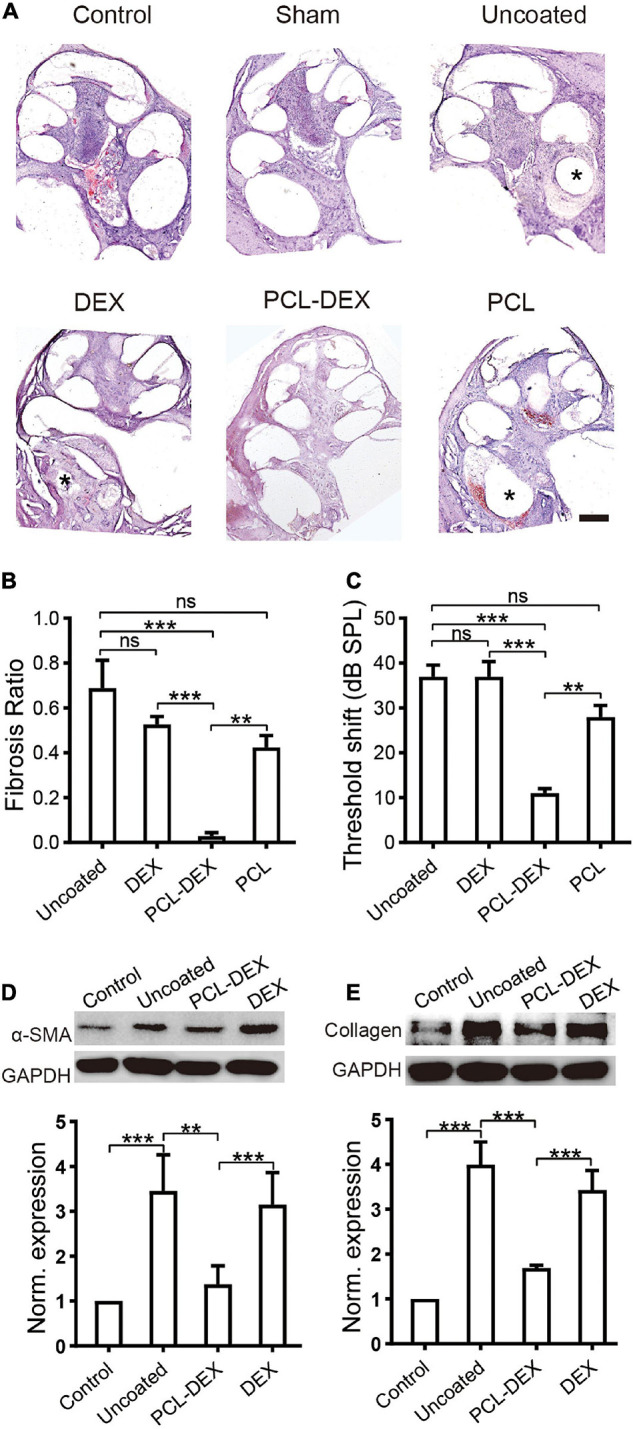
PCL-DEX electrode alleviates fibrosis after CI. **(A)** Optical microscopy images show representative H&E-stained cross sections of the cochlea before (control) and 5 weeks after different implantation procedures. “Sham” for cochlea 5 weeks after sham surgery. “Pristine” for pristine electrode implant. “DEX” for pristine electrode implantation with one dose of DEX applied *via* a round window. “PCL” for implantation with a PCL-coated electrode containing no DEX. “PCL-DEX” for implantation with a PCL-coated electrode containing 20% DEX. Scale bar = 500 μm for all panels. **(B)** The fibrosis ratio 5 weeks after different implant procedures. **(C)** The ABR threshold shift at 2 kHz 5 weeks after different implantation procedures. The data in panels **(B**,**C)** are presented as the mean ± SE. ns: no significant difference, *p* > 0.05; *p < 0.05; **p < 0.01; ***p < 0.001, Student’s *t*-test. *n* = 5 for each group. **(D)** The effects of different implant treatments on the expression of fibroblastic proliferation-related proteins in the cochlea. **(D)** Representative immune bands (upper panel) and normalized expression level (lower panel) of α-SMA in the cochlea before (control) and 5 weeks after different implant procedures. **(E)** Representative immune bands (upper panel) and normalized expression level (lower panel) of type I collagen in the cochlea before (control) and 5 weeks after different implant procedures. “Pristine” for pristine electrode implant. “PCL-DEX” for implantation with a PCL-coated electrode containing 20% DEX. “DEX” for pristine electrode implantation with one dose of DEX applied *via* a round window. The experiment was repeated three times in each group. All datapoints are presented as the mean ± SD. ns: no significant difference, *p* > 0.05; *p < 0.05; **p < 0.01; ***p < 0.001.

Fibrosis was examined in the cochleae at week five after the implantation of a bare silicone electrode (pristine), silicone electrode coated with PCL-DEX, or silicone electrode coated with PCL only. Meanwhile, in a group of bare silicone electrode implantations, one dose of local DEX was applied at insertion to mimic the clinical treatment. As shown by the representative cross sections in Figure 5A, in the pristine, PCL, and DEX groups, large fibrotic tissue around the implants compared to the control and sham groups was observed, and little fibrosis was found in the PCL-DEX group. The quantified data showed that the volume of fibrotic tissue was significantly decreased for PCL-DEX-coated implants (Figure 5B). This coating presented average reductions of 96, 93, and 95% in fibrosis compared with the uncoated, PCL-coated, and one-dose DEX treatment groups, respectively (*p* < 0.001, *p* = 0.0057, *p* < 0.001, respectively, one-way ANOVA). The hearing of the implanted ear was protected by the PCL-DEX coating, as demonstrated by a significantly smaller ABR threshold shift than that of the uncoated, PCL-coated, and one-dose DEX treatment groups (Figure 5C, *p* < 0.001, *p* = 0.0011, *p* < 0.001, respectively, one-way ANOVA). The expression of α-SMA and type I collagen, markers of fibrotic tissue, was measured in the cochleae to further confirm the inhibition of fibrosis by PCL-DEX coating. Significantly lowerα-SMA (Figure 5D, *p* = 0.0013, one-way ANOVA) and type I collagen (Figure 5E, *p* < 0.001, one-way ANOVA) expression was observed in the PCL-DEX-coated implants than in the uncoated implants, while the expression was the same in the local DEX treatment group (*p* > 0.05 for both expression levels, one-way ANOVA).

**FIGURE 6:**
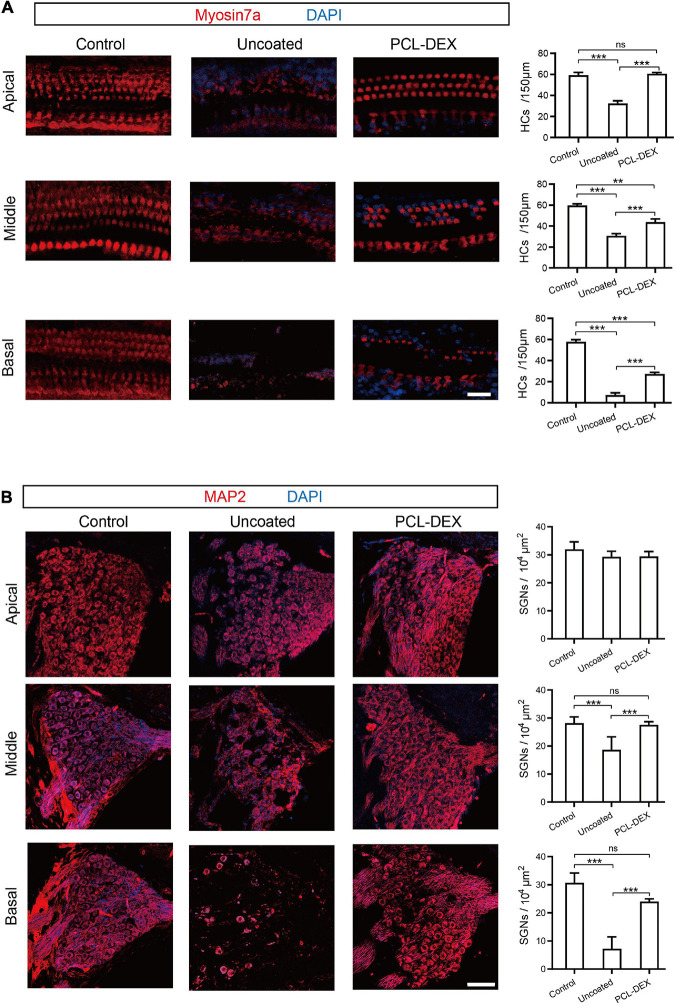
The effects of different implant treatments on HCs and SGNs. **(A)** Representative immunofluorescence staining for myosin 7a (red) as a marker for HC (left panel) and HC density (right panel) at the basal, middle, and apical turns from cross sections of the cochlea before (control) and 5 weeks after implantation with pristine and PCL-DEX electrodes. Scale bar = 20 μm. **(B)** Representative immunofluorescence staining for MAP2 (red) as a marker for SGN (left panel) and SGN density (right panel) at the basal, middle, and apical turns from cross sections of the cochlea before (control) and 5 weeks after implantation with pristine and PCL-DEX electrodes. Scale bar = 30 μm. The data are presented as the mean ± SE. ns: no significant difference, *p* > 0.05, *n* = 3 for each group. **p < 0.01; ***p < 0.001.

The protective effects of the PCL-DEX coating may benefit from less progressive impairment-induced cell loss in hair cells (HCs) and spiral ganglion neurons (SGNs) (Zhou et al., 2020). As shown in Figure 6A, compared with the controls, significant loss of HCs was observed along the whole cochlea at week 5 after the implantation of pristine electrodes (*p* < 0.001 for all three positions, one-way ANOVA). The cell loss at apical segments of the cochlea, at which the electrode did not directly reach, indicates a progressive impairment of HCs in addition to the possible direct trauma of the basal cochlea by implant insertion. This loss of HCs was significantly reduced by the implantation of the PCL-DEX-coated electrode (Figure 6A, *p* < 0.001 for all three positions, one-way ANOVA). The protective effect of the PCL-DEX coating was also observed for SGNs. The densities of SGNs at the middle and basal cochlea were decreased at week 5 after the implantation of pristine electrodes (Figure 6B, *p* < 0.001 for both positions, one-way ANOVA). Such loss of SGNs was alleviated by the PCL-DEX-coated electrode (for both positions *p* < 0.001 vs. pristine, one-way ANOVA), and no significant loss of SGNs was found compared with the controls (*p* > 0.05 vs. controls, one-way ANOVA). These results are consistent with our functional measurements of ABR recordings.

**FIGURE 7:**
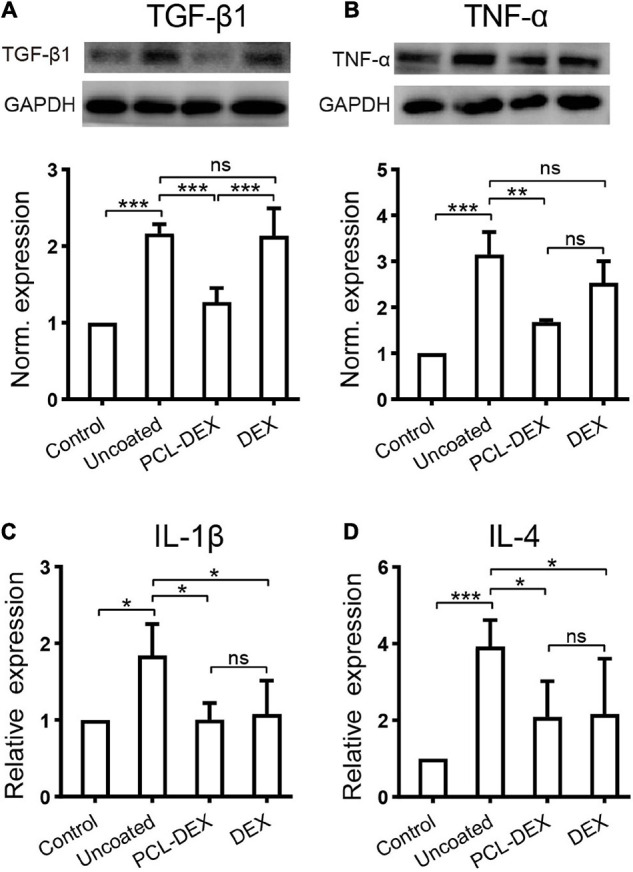
The effects of different implant treatments on the expression of inflammatory factors in the cochlea. **(A)** Representative immune bands (upper panel) and normalized expression level (lower panel) of TGF-β1 in the contralateral cochlea (control) and 7 days after different implant procedures. *n* = 4 for each group. **(B)** Representative immune bands (upper panel) and normalized expression level (lower panel) of TNF-α in the contralateral cochlea (control) and 1 day after different implant procedures. *n* = 4 for each group. **(C)** ELISA shows the normalized expression of IL-1β in cochlear lysates 1 day after different implant procedures. *n* = 4 for each group. **(D)** ELISA shows the normalized expression of IL-4 in cochlear lysates 7 days after different implant procedures. *n* = 5 for each group. “Pristine” for pristine electrode implant. “PCL-DEX” for implantation with a PCL-coated (MW = 36 k) electrode containing 20% DEX. “DEX” for pristine electrode implantation with one dose of DEX applied *via* a round window. The experiment was repeated at least three times in each group. All data points are presented as the mean ± SD. ns: no significant difference, *p* > 0.05; *p < 0.05; **p < 0.01; ***p < 0.001, Student’s *t*-test.

It is of great significance to reveal the possible mechanisms of how the PCL-DEX-coated implant protected SGNs and HCs, reduced fibrotic tissue, and restored hearing. The role of inflammation in the foreign body response resulting in fibrosis and cell death has been well recognized. In the present study, inflammation-related factors were examined to determine the effects of PCL-DEX-coated implants. The western blotting in Figure 7A shows that the expression of TGF-β1 was increased by pristine implants with/without local DEX treatment, indicating an increase in the foreign body response in the cochlea (for both implants, *p* < 0.001 vs. controls, one-way ANOVA). This TGF-β1 increase was significantly reduced by the PCL-DEX coating (*p* < 0.001 vs. pristine and DEX only, one-way ANOVA). TNF-α and IL-1β, two factors in the early inflammation phase, were measured in cochleae by western blotting and ELISA (Figures 7B,C). Compared with the controls, the expression of both factors was increased by the implantation of pristine electrodes (*p* < 0.001 and *p* < 0.05, respectively, one-way ANOVA). The PCL-DEX coating reduced the increase in these factors (*p* < 0.01 and *p* < 0.05, respectively, one-way ANOVA), while local DEX treatment exhibited a weak influence only on IL-1β (*p* < 0.05, one-way ANOVA). IL-4, a factor in the later inflammation phase, was examined 3 days after implantation (Figure 7D). Similar to the effects on TNF-α and IL-1β, the PCL-DEX coating reduced the expression of IL-4 induced by the implant (*p* < 0.05 vs. pristine, one-way ANOVA), and the reduction was more significant than local DEX treatment (*p* < 0.05, one-way ANOVA).

**FIGURE 8:**
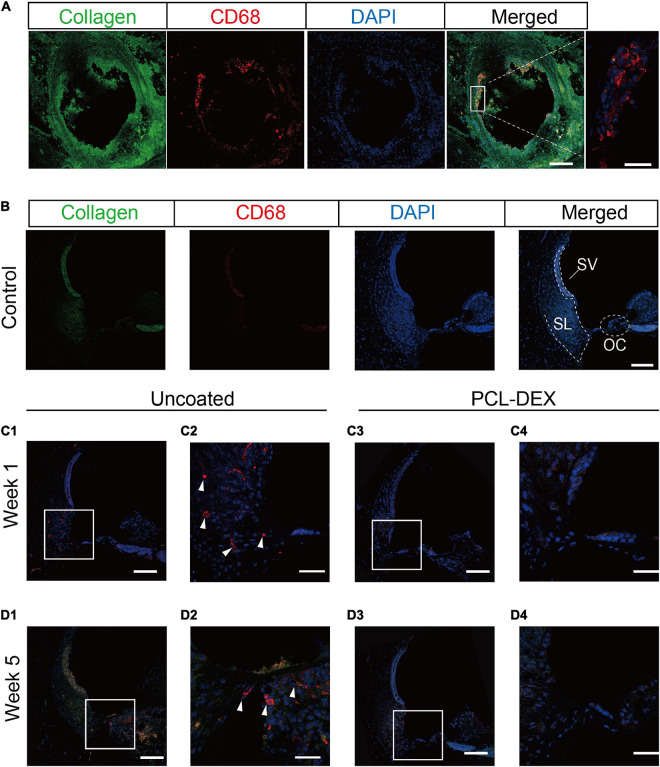
Confocal images of macrophages expressed in the cochlea. **(A)** Representative immunostaining images showing the proliferative tissue in the tympanic scala 5 weeks after implantation. CD68 (red) and type I collagen (green) were evident in the proliferative tissue, especially on the surface of the electrode (magnified view, right panel). Scale bar = 100 and 20 μm for the left and right panels, respectively. **(B)** Representative immunostaining images showing the spiral ligament in the control cochlea. SL, spiral ligament; SV, stria vascularis; OC, organ of Corti. **(C)** Representative immunostaining images showing the spiral ligament of the basal turn in the cochlea implanted with pristine or PCL-DEX electrodes for 1 week **(C1,C3)** and 5 weeks **(D1,D3)**. **(C2,C4,D2,D4)** are magnified views of panels **(C1,C3,D1,D3)**, respectively, and solid white arrows indicate macrophages positive for CD68 (red). Scale bar = 100 μm.

For the foreign body response, macrophages play a key role in inflammation and the formation of fibrotic tissue. As shown in the cochlear cross section 5 weeks postimplantation of the uncoated electrode, CD68-marked macrophages were found in the fibrotic tissue around the pristine implant (Figure 8A). However, in the intact contralateral cochlea, no macrophages were observed in the OC, stria vascularis (SV), and spiral ligament (SL) regions (Figure 8B). To investigate how macrophages invade the cochlea after cochlear implantation, we examined the distribution of macrophages in the cochlea. CD68 staining 1 week postimplantation showed that macrophages first appeared in the SL regions in the uncoated electrode-implanted cochlea (Figures 8C1,C2), while macrophages were rarely observed in the PCL-DEX-implanted cochlea (Figures 8C3,C4). At 5 weeks postimplantation of the uncoated implant, macrophages were observed in fibrotic tissue, while macrophages were also found in the SV, OC, and SL regions (Figures 8D1,D2). The PCL-DEX-coated implant showed much less fibrotic tissue in the scala tympani. Moreover, very few macrophages were observed in the SV, OC, and SL regions compared to the uncoated implant (Figures 8D3,D4). These data indicate that the PCL-DEX coating resulted in fewer activated macrophages recruited to the cochlea after implantation.

## Discussion

In mammal’s inner ear, sound wave is transformed into electrical signals by cochlear hair cells (Guo et al., 2019; Liu W. et al., 2019; Qi et al., 2019, 2020; Tan et al., 2019; He et al., 2020; Zhang et al., 2020; Chen et al., 2021) and then these signals are transduced to auditory cortex by spiral ganglion neurons (Guo et al., 2016, 2021; Fang et al., 2019; Liu Y. et al., 2019; Zhao et al., 2019; Guo R. et al., 2020; Liu et al., 2021). Cochlear implantation can perfectly replace the function of hair cells, and thus is the major treatment for severe sensorineural hearing loss (Guo J. H. et al., 2020). However, fibrotic tissue after cochlear implantation (O’Malley et al., 2017) may lead to residual hearing loss (Reiss et al., 2015) in patients. In the present study, hearing impairment and fibrosis after CI were also observed in our animal models (Figures 1A–C). Our results (Figure 2) together with related studies have indicated that fibrotic tissue is composed of myofibroblasts and extracellular matrix (for example, type I collagen) (Jia et al., 2016). Since individual differences exist, the level of fibrosis but not implantation time plays a key role in the prognosis after CI. The greater the fibrotic volume in the cochlea, the worse the residual hearing loss (Figures 1D–F). Consistent with the present study, individual differences in humans during fibrotic progression after CI have been found, and no significant correlation between level of fibrosis and implantation time was found (Ishai et al., 2017). These clinical problems are expected to be solved with long-term release of dexamethasone with a PCL-coated electrode. Corresponding to the location of the implant that is vulnerable to direct mechanical damage from the electrodes, hearing thresholds at high frequencies did not vary significantly with changes in the level of fibrosis. However, hearing thresholds at low frequency (2 kHz) vary as a function of the level of fibrosis, where the apical turn of the cochlea far away from the implant. Two reasons may involve in the hearing loss. First, fibrosis after CI may attenuate the vibration of perilymph. Secondly, the release of profibrotic factors and inflammation producers during the progression of fibrosis causes damage to hair cells and SGNs (Figure 6). In the present study, dexamethasone was selected to alleviate fibrosis in cochlear implantation. Long-term release of dexamethasone with a PCL-coated electrode alleviated fibrosis and improved hearing function after CI compared with the uncoated electrode (Figure 5). Hair cells and spiral ganglion cells were well preserved by the PCL-DEX electrode (Figure 6).

Polycaprolactone is an attractive biomedical polymer with slow biodegradability, good biocompatibility, good drug permeability, and relatively low production cost (Wei et al., 2009). PCL has been used in basic research and approved for some clinical use, including wound healing, tissue regeneration, and drug control delivery systems (Liang et al., 2020). Although it has not been applied to intracochlear administration, PCL did not affect the survival of cells and or the microenvironment in the cochlea (Figures 4E,F). Moreover, abundant apertures that were homogenic and small in size were instrumental in the steady and sustained release of DEX (Figure 3C), that was supported by our *in vitro* data (Figure 4). Clinically, glucocorticoids are administered systemically or locally to treat fibrosis following implantation (Stathopoulos et al., 2014). Due to individual differences and other reasons, the current clinical efficacy varies and is not significant. Systemic and local dexamethasone delivery cannot be continued for a long time because of side effects and technical difficulties. After discontinuation of DEX administration, fibrosis progresses and the effectiveness of cochlear implantation decreases, suggesting that sustained interventions should be carried out (Mamelle et al., 2017). Since fibrotic reactions continuously develop, fibrosis will further develop after discontinuation of DEX (Liu et al., 2015). PCL was reported to achieve controlled release with a relatively low release rate (Song et al., 2014). Our PCL-DEX electrodes controlled drug delivery system managed to release DEX at a stable and effective concentration for a much longer time. The effectiveness of our PCL-DEX electrode lies in the fact that it can reach all cochlear locations that electrodes reach, and it can persist longer for dexamethasone to work (Figures 4A–D). Implied by our experimental evidence, the PCL-DEX electrode had better efficacy than a single dose of DEX (Figure 5).

Drug elution from the cochlear implant has been explored previously using a mini-osmotic pump or drug loaded in a gel surrounding the implant (Mamelle et al., 2017; Xu et al., 2018). Other reported drug delivery systems are not able to achieve long-term continuous administration or maintain a therapeutic level of drug concentration for a long time. Some reported drug delivery systems in the cochlea lasted only approximately 3 weeks after surgery. In our study, the PCL-DEX-coated electrode has the advantage of high drug loading efficiency compared with other methods, such as capsuling drugs or drug pumping (Richardson et al., 2009). Additionally, when DEX was carried by PCL into the scala tympani, the DEX was released directly into the cochlea, and DEX release lasted longer, resulting in a decrease in fibrosis. Our findings support the hypothesis that coated implant in the cochlea enables sustained release of the drug reduces fibrosis caused by postoperative implantation trauma. In addition, the PCL coating can also carry drugs other than DEX to target fibrosis in the cochlea (Puga et al., 2012; Zhao et al., 2012).

This treatment also allows us to further explore the mechanism of inhibiting fibrosis better and find more precise therapeutic targets, enabling more specific and accurate treatment. In the early stage after CI, the expression of TNF-α and IL-1β increased in the cochlea (Yamahara et al., 2018). In the advanced stage of fibrosis, the expression of IL-4 and TGF-β1 increased and the TGF-β1was observed in the fibrous tissue around the electrode implanted (Figures 2, 7). Fibrosis-associated mechanisms have been elucidated in various organs, such as the liver, kidney, lung, heart, and skin, but they need to be further investigated in the cochlea. IL-1β, TNF-α, and IL-4 are critical proinflammatory contributors to the pathogenesis of inflammation and fibrosis (Weiskirchen et al., 2019). Significant increases in expression were observed for inflammatory genes after cochlear implantation (Zhang et al., 2015; Eshraghi et al., 2019). TGF-β1 plays a leading role in the process of fibrosis in cochlear implantation by stimulating myofibroblast differentiation and collagen synthesis and deposition (Bas et al., 2020). The PCL-DEX electrode reduced the expression of TNF-α, IL-1β, IL-4, and TGF-β1 (Figure 7) and thus reduced myofibroblast infiltration and decreased collagen deposition (Figure 5). The PCL-DEX electrode achieved a therapeutic effect by blocking the TGF-β1 signaling pathway.

Local macrophage is a main source of TGF-β1 during fibrosis (Chung et al., 2018; Tang et al., 2019). Macrophages and foreign body giant cells (FBGCs) were found in a previous pathologic study of the temporal bone after cochlear implantation (Bas et al., 2020). Previous studies have shown that the activation of myofibroblasts and the production of extracellular matrix induced by the production of TGF-β1 by macrophages are key links in the process of fibrosis in other organs (Wynn and Ramalingam, 2012). After uncoated electrode implantation, macrophages migrate to the surface of the electrode (Bellamri et al., 2019). Foreign body reactions can lead to the degradation of CI biomaterials. It is presumed that these biomaterials are subject to mediators of degradation released by macrophages and FBGCs and undergo phagocytosis in an attempt to clear the debris (Liu et al., 2018; Yan et al., 2018; Wu et al., 2020). FBGCs were seen at the interface between the electrode and the fibrous capsule (Figure 8A). Correspondingly, the number of macrophages increased after uncoated electrode implantation. PCL-DEX electrodes carried more DEX and had a longer DEX release time than other approaches (Lehner et al., 2019), and the number of macrophages decreased after PCL-DEX electrode implantation (Figure 8). Together, these data suggest that PCL-DEX electrodes reduce fibrosis by inhibiting the proliferation of macrophage cells, the secretion of fibrosis-related factors, and inhibiting the proliferation of myofibroblasts into the scala tympani. Therefore, the coating of PCL-DEX is beneficial to improve the long-term effect after cochlear implantation.

## Data Availability Statement

The original contributions presented in the study are included in the article/supplementary material, further inquiries can be directed to the corresponding author/s.

## Ethics Statement

The animal study was reviewed and approved by Institutional Animal Care and Use Committee (IACUC) of Southern Medical University.

## Author Contributions

HZ and JT designed the experiments. DM provided technical assistance with preparation and performance detection on the polycaprolactone coated electrode. DC, JT, and HZ wrote the manuscript. DC, YL, JP, AC, and MX performed experiments. All authors reviewed, edited, and approved the final manuscript.

## Conflict of Interest

The authors declare that the research was conducted in the absence of any commercial or financial relationships that could be construed as a potential conflict of interest.

## Publisher’s Note

All claims expressed in this article are solely those of the authors and do not necessarily represent those of their affiliated organizations, or those of the publisher, the editors and the reviewers. Any product that may be evaluated in this article, or claim that may be made by its manufacturer, is not guaranteed or endorsed by the publisher.
